# Epidemiological and clinical characteristics of immunocompromised patients infected with *Pneumocystis jirovecii* in a twelve-year retrospective study from Norway

**DOI:** 10.1186/s12879-021-06144-1

**Published:** 2021-07-07

**Authors:** Stine Grønseth, Tormod Rogne, Raisa Hannula, Bjørn Olav Åsvold, Jan Egil Afset, Jan Kristian Damås

**Affiliations:** 1 Department of Clinical and Molecular Medicine, Faculty of Medicine and Health Sciences, NTNU - Norwegian University of Science and Technology, NO-7491 Trondheim, Norway; 2 Department of Circulation and Medical Imaging, NTNU, Trondheim, Norway; 3 Department of Infectious Diseases, St. Olavs hospital, Trondheim University Hospital, Trondheim, Norway; 4 K.G. Jebsen Center for Genetic Epidemiology, Department of Public Health and Nursing, NTNU, Trondheim, Norway; 5 HUNT Research Center, Department of Public Health and Nursing, NTNU, Levanger, Norway; 6 Department of Endocrinology, St. Olavs hospital, Trondheim University Hospital, Trondheim, Norway; 7 Department of Medical Microbiology, St. Olavs hospital, Trondheim University Hospital, Trondheim, Norway; 8 Centre of Molecular Inflammation Research, NTNU, Trondheim, Norway

**Keywords:** *Pneumocystis jirovecii*, PCP, Pneumonia, Immunosuppression, Immunocompromised

## Abstract

**Background:**

*Pneumocystis* pneumonia (PCP) severely menaces modern chemotherapy and immunosuppression. Detailed description of the epidemiology of *Pneumocystis jirovecii* today is needed to identify candidates for PCP-prophylaxis.

**Methods:**

We performed a 12-year retrospective study of patients with *P. jirovecii* detected by polymerase chain reaction in Central Norway. In total, 297 patients were included. Comprehensive biological, clinical and epidemiological data were abstracted from patients’ medical records. Regional incidence rates and testing trends were also assessed.

**Results:**

From 2007 to 2017 we found a 3.3-fold increase in testing for *P. jirovecii* accompanied by a 1.8-fold increase in positive results. Simultaneously, regional incidence rates doubled from 5.0 cases per 100,000 person years to 10.8. A majority of the study population had predisposing conditions other than human immunodeficiency virus (HIV). Hematological (36.0%) and solid cancers (25.3%) dominated. Preceding corticosteroids were a common denominator for 72.1%. Most patients (74.4%) presented with at least two cardinal symptoms; cough, dyspnea or fever. Main clinical findings were hypoxia, cytopenias and radiological features consistent with PCP. A total of 88 (29.6%) patients required intensive care and 121 (40.7%) suffered at least one complication. In-hospital mortality was 21.5%. Three patients (1.0%) had received prophylaxis.

**Conclusions:**

*P. jirovecii* is re-emerging; likely due to increasing immunosuppressants use. This opportunistic pathogen threatens the life of heterogenous non-HIV immunosuppressed populations currently at growth. Corticosteroids seem to be a major risk factor. A strategy to increase prophylaxis is called for.

**Supplementary Information:**

The online version contains supplementary material available at 10.1186/s12879-021-06144-1.

## Background

Iatrogenic immunosuppression represents a double-edged sword in the era of modern medicine. While improving the lives and life expectancy of individuals living with chronic autoimmune disorders, organ transplants or cancers, immunosuppressive drugs also increase the risk of opportunistic infections [1]. *Pneumocystis* pneumonia (PCP), primarily associated with the human immunodeficiency virus (HIV) epidemic in the 1980’s, represents one of these [2]. PCP is often severe and mortality rates are high, especially in non-HIV immunocompromised patients [3].

Adequate identification of patients with PCP is challenging and relies on clinical suspicion. The manifestations of PCP are non-specific and include cough, dyspnea, fever and hypoxemia, in addition to constitutional symptoms like malaise and weight loss [4]. Therefore, PCP is often mistaken for pneumonia of another bacterial, viral or fungal etiology, malign processes, sarcoidosis and non-infectious interstitial pneumonitis [5], or in our experience pulmonary embolism. Polymerase chain reaction (PCR) for molecular detection of microorganisms and high-resolution CT-scans are of essential value in this context, particularly among immunocompromised hosts [5]. In the case of *Pneumocystis jirovecii*, distinction between colonization and PCP poses a particular challenge as the risk factors are overlapping [6]. Moreover, the high sensitivity of PCR for *P. jirovecii* detection compromises its specificity for PCP diagnosis [5]. In contrast, the sensitivity of microscopic visualization of cysts or trophic forms is limited, specifically among non-HIV individuals and on specimens from the upper respiratory tract due to low fungal inoculums and reduced chance of detection [3].

Knowledge and awareness about iatrogenic risk factors are required for considering PCP as a differential diagnosis and for prescribing prophylaxis to susceptible individuals. Immunosuppressants associated with PCP include corticosteroids, a wide spectrum of chemotherapeutic regimens, synthetic steroid-sparing drugs, and modern biological immunomodulators such as anti-tumor necrosis factor [2].

In Norway, PCP is not a notifiable disease unless it occurs in an HIV-infected individual as a manifestation of acquired immunodeficiency syndrome (AIDS) [7]. Therefore, the incidence in immunocompromised non-HIV patients, their host characteristics and the burden across HIV-status are unknown. Due to extensive use of immunosuppressants susceptible populations are currently at growth [2]. Herein we describe epidemiological and clinical characteristics among immunocompromised patients assessed for PCP in a 12-year retrospective study.

## Methods

### Setting

Our study was based on data from St. Olavs hospital, Trondheim University Hospital, which is the only tertiary referral hospital in Central Norway. The health region offers services to approximately 700,000 inhabitants representative of the national population [8, 9]. Until 2017, St. Olavs hospital had the only microbiology laboratory conducting *P. jirovecii* diagnostics in the region. All patients from central Norway with *P. jirovecii* detected in one or more respiratory samples by PCR in St. Olavs hospital between 2006 and 2017 were identified and linked to their respective medical records. Only primary episodes were included. All subjects 16 years or older at the time of testing were eligible. Alive patients were included on the basis of informed consent in 2018. There were no minors among these. The need for consent from next of kin or legal guardian of deceased patients was waived by the ethics committee.

### Patient characteristics and data collection

We retrospectively reviewed the medical records of the study population and extracted comprehensive epidemiological, laboratory and clinical data. The software Epi Info™ (version 7.2.2.6; Centers for Disease Control and Prevention, Atlanta, GA, USA) was used to record patient data. The number and severity of combined comorbid conditions were assessed according to the Charlson weighted comorbidity index [10]. Corticosteroid exposure pattern 60 days preceding presentation was categorized as daily, intermittent or none. In case of ongoing corticosteroid intake on the date of *P. jirovecii* detection, the daily dose was converted into the equivalent in methylprednisolone expressed as milligrams per day and the median among users was calculated. Antimicrobial treatments administered after the detection of *P. jiroveci*, regardless of etiological indication, were also registered. Treatment and documented complications occurring in association with hospitalization were also recorded. Date of death was ascertained through linkage with the Norwegian Population Register through the end of June 2018 for sufficient follow-up.

### Samples and definition of PCP

Diagnostic respiratory specimens included bronchoalveolar lavage fluid, lung biopsies, sputum samples, induced sputum, nasopharyngeal swabs and tracheal aspirates. In two patients, definitive detection of *P. jirovecii* was performed post-mortem upon autopsy. In cases where multiple respiratory samples were taken from a patient, those from the lower respiratory tract, primarily bronchoalveolar lavage fluid, were preferred due to their superior diagnostic yield in the setting of PCP.

The PCR analysis was performed as an in-house real-time PCR targeting the beta-tubulin gene of *P. jirovecii*, as previously described [11], with some modifications. PCR reagents and instruments used varied through the study period, but all changes were validated to ensure equal quality. The laboratory participated in a *Pneumocystis jirovecii* pneumonia (PCP) DNA EQA Programme (QCMD) from 2012. Semiquantitative estimation of fungal loads was performed on positive samples and results were reported with cycle threshold (*C*_*T*_) values. *C*_*T*_ values are defined as the replicated number at which the fluorescence generated within a reaction crosses the fluorescens threshold line [12]. Accordingly, a low *C*_*T*_ value corresponds to a high fungal burden and vice versa*.* Microscopy (direct immunofluorescence (DIF) was performed with MONOFLUO *Pneumocystis jirovecii* IFA Test Kit #32515 (Bio-Rad). The assay was in use at the laboratory until 2017, mainly on samples resulting positive by PCR whenever positive controls were available. To discriminate cases of PCP (PCP^+^) from colonization (PCP^−^), we applied retrospective case-criteria in line with the European Conference on Infections in Leukaemia (ECIL) guidelines [13] with the available data. According to previous studies, we considered that *C*_*T*_ values above 35 corresponded to colonization and not overt PCP [12, 14] regardless of the host’s HIV status. Thus, the criteria for PCP^+^ among our PCR-positive cohort were i) positive DIF and/or ii) *C*_*T*_ value below 36. Patients with *C*_*T*_ values above 35 and negative or missing DIF result were considered colonized with *P. jirovecii* (i.e., PCP^−^). Patients with missing *C*_*T*_ value and negative or missing DIF result were classified as “undetermined PCP-status”.

### Estimation of incidence rates

To estimate regional incidence rates, we accessed the online databank of Statistic Norway to retrieve the total number of people 16 years or older living in Central Norway during the study period [15]. These counts represented the denominators in our yearly incidence rate estimates. PCR detection of *P. jirovecii* was introduced at St. Olavs hospital, our referral laboratory, in late 2006. Thus, estimates were calculated for 2007 to 2017. In 2017, Molde hospital, a local hospital in the health region, established PCR-testing for *P. jirovecii* too. For completeness, individuals with a positive result at the laboratory in Molde hospital in 2017 were included in the regional incidence estimates for that year.

### Statistics

Continuous quantitative variables are presented as medians with second (q_1_) and third (q_3_) quartiles or means with standard deviation (+ SD). Discrete variables are given as absolute numbers (percentages). Incidence rate estimates are given with 95% confidence intervals. All analyses were performed using Microsoft Excel (version 16.4; Microsoft Corporation, Redmond, WA, USA) or STATA/MP (version 15.1; College Station, TX, USA).

## Results

### Description of study population

**Fig. 1:**
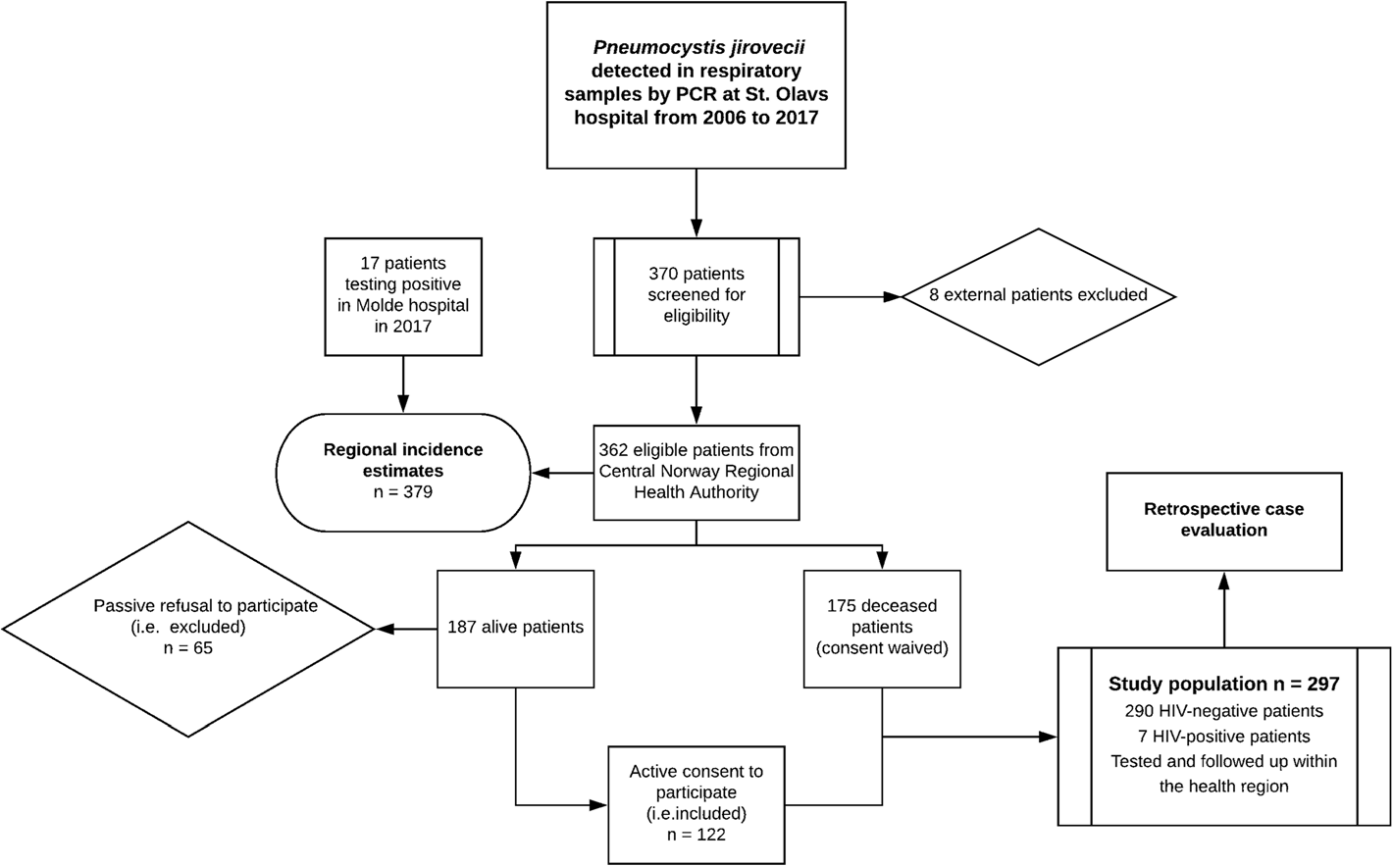
Flowchart. Flowchart of the study population. Adult patients tested in the regional referral laboratory of St. Olavs hospital and followed up in hospitals comprised by the health region of Central Norway were included in the final study population. Recruitment of alive patients required active consent in accordance with the resolution of the regional ethical committee. Molde hospital, a local hospital in the health region, established PCR detection for *P. jirovecii* too in 2017. Individuals resulting positive there were included in the regional incidence estimates for 2017, but not in the study population. PCR, polymerase chain reaction

A final 297 patients (117 F, 180 M) from Central Norway whose samples tested positive for *P. jirovecii* by PCR in the microbiology laboratory of St. Olavs hospital, were included in the study cohort (Fig. 1). The median patient age was 66 years and a majority (60.6%) of the patients were male. Each patient was classified with respect to their principal immunosuppressive condition associated with *P. jirovecii* and PCP development (Table 1). Hematological malignancies were the major predisposing conditions, present in more than one third of the patients (36.0%), with non-Hodgkin lymphomas appearing most frequently of these (51.4%). The second largest subpopulation was constituted by patients with solid tumors (25.3%). Therein, lungs including pleural membranes were the most common origin of the primary tumor (36.0%).

**Table 1:** Characterization of the study population; 297 patients with positive PCR for *Pneumocystis jirovecii*

**Male sex, n (%)**	180 (60.6)
**Ever smoking, n (%)**	162 (54.5)
**Age (years), median, (q**_**1**_**-q**_**3**_**)**	66 (59–74)
**Immunosuppressive conditions, n (%)**	
***Hematological malignancies***	**107 (36.0)**
Non-Hodgkin’s lymphoma	55 (18.5)
Chronic leukemia	17 (5.7)
Plasma cell disease	15 (5.1)
Acute leukemia	11 (3.7)
Hodgkin’s lymphoma	9 (3.0)
***Solid tumors***	**75 (25.3)**
Lung including pleural membranes	27 (9.1)
Breast	14 (4.7)
Genitourinary tract	14 (4.7)
Gastrointestinal tract	9 (3.0)
Other primary tumor^a^	11 (3.7)
***Immunological disorders***	**46 (15.5)**
Rheumatoid arthritis	16 (5.4)
Connective tissue disorders and vasculitidies	15 (5.1)
Miscellaneous^b^ disorders	15 (5.1)
***Solid organ transplantations***	**37 (12.5)**
Kidney	31 (10.4)
Heart, lung	6 (2.0)
***Chronic lung diseases***	**18 (6.1)**
Interstitial lung disease or sarcoidosis	11 (3.7)
Chronic obstructive pulmonary disease	7 (2.4)
***HIV-infection***	**7 (2.4)**
***Other***^***c***^	**7 (2.4)**
**Comorbid conditions, n (%)**	
Hypertension	92 (31.0)
Cardiovascular disease	83 (27.9)
Chronic pulmonary disease	52 (17.5)
Diabetes mellitus type 1 or 2	44 (14.8)
Solid tumor	30 (10.1)
Chronic kidney disease	38 (12.8)
Congestive heart failure	18 (6.1)
Rheumatic disease	12 (4.0)
Hematological malignancy^d^	13 (4.4)
Chronic liver disease	4 (1.4)
**Charlson comorbidity index, n (%)**	
< 4	47 (15.8)
4–6	132 (44.4)
> 6	118 (39.7)

*Abbreviations*: *AIHA* autoimmune hemolytic anemia, *ITP* immune thrombocytopenic purpura, *PCR* polymerase chain reaction

^a^Other primary tumors include brain tumors (i.e., astroglioma, meningioma), nasopharyngeal carcinoma, melanoma, adrenal gland tumor, sarcoma, and mesothelioma

^b^Miscellaneous immunological disorders include hematological disorders (ITP, AIHA), skin disorders, uveitis, inflammatory diseases of gastrointestinal tract and arthritidies other than rheumatoid arthritis

^c^Other immunosuppressive conditions include one patient with statin-induced myositis, one patient with common variable immunodeficiency and four patients with no established disorder at the time of presentation

^d^In 13 patients, hematological malignancy was not considered the primary immunosuppressive condition nor indication for immunosuppression but rather comorbidity

Behind malignancies, various non-HIV conditions appeared with decreasing frequencies, including immunological disorders (15.5%), solid organ transplantation (12.5%) and chronic lung disease (6.1%).

In our cohort, only a minority of seven patients (2.4%) presented with PCP in the context of HIV-infection. One patient from South-East Asia died pre-hospitally and resulted HIV positive in the referral hospital post-mortem. Four patients were unaware of their HIV-status and were naïve to anti-retroviral therapy, while the remaining two were not adherent to their anti-retroviral regimen.

Previous known comorbidities were present in 71.4% of patients, with hypertension being the most prevalent (Table 1). Moreover, the Charlson comorbidity index was skewed towards higher values, indicating an old study population and high degree of comorbidities overall.

### Premorbid iatrogenic immunosuppression, chemotherapy and corticosteroid exposure

Nearly all of the non-HIV study subjects had received immunosuppressants or chemotherapy before assessment for PCP. Ongoing drug regimens prescribed in vicinity to presentation were registered and categorized (Table 2).

**Table 2:** Premorbid immunosuppression, chemotherapy and corticosteroid exposure among 297 patients with positive PCR for *P. jirovecii*

**Immunosuppression/chemotherapy regimens at presentation, n (%)**	
Chemotherapy for hematological malignancy with adjuvant corticosteroids	67 (22.6)
Corticosteroids in monotherapy	44 (14.8)
Graft rejection prophylaxis after solid organ transplantation	36 (12.1)
Chemotherapy for solid malignancy with adjuvant corticosteroids	33 (11.1)
DMARDs with adjuvant corticosteroids	22 (7.4)
Chemotherapy for solid malignancy	16 (5.4)
Chemotherapy for hematological malignancy	12 (4.0)
Corticosteroids and other immunosuppressants^a^	8 (2.7)
DMARDs in monotherapy	5 (1.7)
Prophylaxis or treatment for GVHD after allogenic stem cell transplantation	3 (1.0)
Other combinations^b^	2 (0.7)
None	49 (16.5)
**Systemic corticosteroid exposure last 60 days prior to presentation, n (%)**	
Daily	125 (42.1)
Intermittent	91 (30.6)
No exposure to systemic corticosteroids	79 (26.6)
No information	2 (0.7)
**Corticosteroid daily dosage in mg methylprednisolone at presentation,** ***n*** **= 292**	
Median the day of *P. jirovecii* detection (q_1_-q_3_), *n* = 146	8 (4–20)
Minimum, maximum	0,120
**Indications for corticosteroid administration among exposed**^**c**^**, n (%)**	
Immunosuppression for immunological disorders or graft rejection prophylaxis	99 (46.3)
Chemotherapy	75 (35.0)
Anti-emesis and other oncological indications^d^	51 (23.8)
Peritumoral oedema in primary and secondary intracranial tumors	16 (7.5)
Hematological and solid malignancies complicated by AIHA or ITP	9 (4.2)

*Abbreviations*: *AIHA* autoimmune hemolytic anemia, *DMARDs* disease-modifying anti-rheumatic drugs, *GVHD* graft-versus-host disease, *ITP* immune thrombocytopenic purpura

^a^Other immunosuppressants include mycophenolate, azathioprine, cyclophosphamide, calcineurin- and mTOR-inhibitors, cyclosporine and hydroxychloroquine

^b^Other combinations of immunosuppressive regimens include one patient receiving graft rejection prophylaxis for solid organ transplantation in combination with chemotherapy for hematological malignancy with adjuvant corticosteroids and one patient receiving azathioprine for vasculitis, respectively

^c^214 patients (72.1%) had known exposure to systemic corticosteroids last 60 days prior to presentation, and proportions are expressed with 214 as denominator. In some cases, corticosteroids were prescribed for more than one indication

^d^Other oncological indications include peritumoral oedema for patients with extracranial tumors, corticosteroids in combination with radiotherapy, vena cava superior syndrome, medulla compression etc.

As expected, the regimens reflected the underlying conditions, namely the etiology for iatrogenic immunosuppression, with chemotherapy with adjuvant steroids for hematological malignancy, being the most common category (22.6%).

Notably, 72.1% had been exposed to systemic corticosteroids in the 2 months preceding evaluation for PCP with a median dose in methylprednisolone of 8 (q_1_-q_3_ 4–20) milligram per day among users the day of *P. jirovecii* detection (*n* = 146). Given the high prevalence of systemic corticosteroid usage, we went on to investigating the indications for prescription among the exposed (*n* = 214), though some patients had multiple indications. Here immunosuppression for immunological disorders and graft rejection prophylaxis had the highest occurrence (46.3%), followed by systemic corticosteroids as chemotherapeutic agents (35.0%), and other oncological indications combined (31.3%), indicating widespread application in treatment of cancer patients in general.

A subpopulation of 49 patients (16.5%), including the seven HIV-positive patients, were not being prescribed immunosuppressant or receiving chemotherapy at presentation. However, 29 of the non-HIV patients in this group (69.0%) had received iatrogenic immunosuppression, chemotherapy or both the last 5 years.

Only three patients (1.0%) were receiving primary PCP-prophylaxis at presentation.

### Clinical, biological and radiological features

All but six patients presented with at least one cardinal symptom of pneumonia; cough, dyspnea, or fever prior to detection of *P. jirovecii* in a respiratory sample. The remaining patients reported reduced general condition, had abnormal findings on their physical or radiographic examinations. Clinical characteristics are summarized in Table 3.

**Table 3:** Clinical characteristics, management and outcome among 297 patients with positive PCR for *Pneumocystis jirovecii*

**Symptoms at baseline, n (%)**	
Dyspnea	219 (73.7)
Fever	214 (72.1)
Cough	169 (56.9)
Two symptoms	221 (74.4)
Three symptoms	92 (31.0)
**Objective baseline findings and biochemistry, median (q**_**1**_**-q**_**3**_**)**	
Oxygen saturation, % (*n* = 285)^a^	89 (85–93)
Hemoglobin, g/dl (*n* = 289)	10.7 (9.7–11.7)
Leukocyte count, × 10^9^/L (*n* = 287)	7.2 (4.3–10.1)
Neutrophil count, × 10^9^/L (*n* = 224)	5.0 (3.0–7.7)
Lymphocyte count, × 10^9^/L (*n* = 152)^b^	0.6 (0.4–1.1)
Albumin, g/L (*n* = 207)	32 (26–36)
Lactate dehydrogenase, U/L (*n* = 165)	293 (224–390)
**Radiological findings, n (%)**	
Remarks on chest X-ray (*n* = 254)	205 (80.7)
Nodular, linear and/or patchy opacities	219 (86.2)
Focal infiltrates	30 (11.8)
Consolidations	11 (4.33)
Remarks on thoracic CT (*n* = 247)	242 (98.0)
Ground glass opacities	188 (76.1)
Thickening of interstitial septa	69 (27.9)
Infiltrates	53 (21.5)
Consolidations	44 (17.8)
Lymphadenopathy	41 (16.6)
Bronchiectasis	18 (7.3)
Three-in-bud sign	16 (6.5)
Cysts	12 (4.9)
**Management and complications, n (%)**	
Receiving PCP-directed treatment	261 (87.9)
Antimicrobials for other pathogens^c^	176 (59.3)
Transferred to an ICU	88 (29.6)
Receiving ventilation support	88 (29.6)
Invasive and/or invasive and non-invasive	50 (16.8)
Non-invasive only	38 (12.8)
Developing complications	121 (40.7)
Respiratory failure/ARDS	83 (27.9)
Superinfection	50 (16.8)
Hemodynamic failure	37 (12.5)
Renal failure	33 (11.1)
Pneumothorax	7 (2.4)
**Outcome, n (%)**	
In-hospital mortality	64 (21.5)
Cumulative all-cause mortality	
30-days	60 (20.2)
90-days	97 (32.7)
180-days	116 (39.1)

*Abbreviations*: *ARDS* acute respiratory distress syndrome, *CT* computed tomography, *ICU* intensive care unit, *PCP Pneumocystis* pneumonia, *PCR* polymerase chain reaction

^a^68 patients received supplemental oxygen when oxygen saturation was measured

^b^Lymphopenia (< 1.0 lymphocytes × 10^9^/L) was present among 108 patients (71.1%) with retrievable lymphocyte counts

^c^163 patients received antibiotics, 56 patients received antifungals, 25 patients received antivirals other than anti-retrovirals and four patients received anti-tuberculous drugs

A selection of objective manifestations and laboratory results are also presented though they were not retrievable for all patients. Decreased oxygen saturation (< 95%) was a common baseline finding, documented in 215 (72.4%) patients on presentation. Differential blood counts were incomplete overall, but therein lymphopenia (< 1.0 ×  10^9^ cells/L) dominated (108 of 152 patients; 71.1%) whilst severe neutropenia (< 0.5 × 10^9^ cells/L) appeared infrequently (6 of 224 patients; 2.7%).

Plain chest radiography was performed in 254 cases, and abnormalities were noted in 219 (86.2%), with interstitial (nodular, linear or patchy) opacities being the most frequent features. Among 247 patients undergoing thoracic CT, 242 (98.0%) manifested abnormalities. Ground glass opacities were present in 73.7% of the cases, followed by thickening of interstitial septa (26.7%), both suggestive signs of PCP, but not pathognomonic.

### Microbiological results and classification of PCP-status

**Fig. 2:**
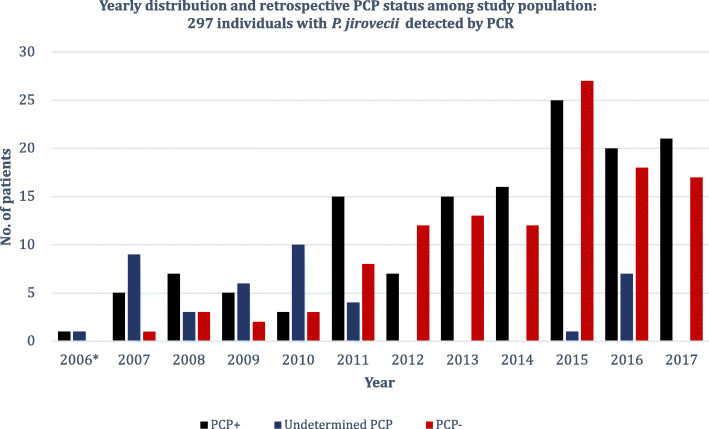
*Pneumocystis* pneumonia (PCP)-status by study year. Yearly distribution of patients with **I)** PCP (PCP^+^) based on i) positive direct immunofluorescence and/or ii) *C*_*T*_ value below 36 (black columns; *n* = 140, 47.1%), **II)** presumed colonization (PCP^−^) not fulfilling the criteria for PCP (red columns; *n* = 116, 39.1) and **III**) “undetermined PCP-status”; patients without information about *C*_*T*_ value and negative or missing DIF result (dark blue columns; *n* = 41, 13.8). Criteria were applied in retrospect. *The study period was from 2006 to 2017, though PCR was introduced in late 2006. *C*_*T*_, cycle threshold; DIF, direct immunofluorescence microscopy; PCP, *Pneumocystis* pneumonia; PCR, polymerase chain reaction

A majority of the patients within our cohort underwent bronchoalveolar lavage for microbiological diagnostics (*n* = 234, 78.8%), followed by sputum (*n* = 44, 14.8%), induced sputum (*n* = 9, 3.0%), tracheal aspiration (*n* = 5, 1.7%), biopsy upon autopsy (*n* = 2, 0.7%), nasopharyngeal sampling (*n* = 2, 0.7%) and lastly transbronchial biopsy (*n* = 1, 0.3%). DIF microscopy for *P. jirovecii* was performed on respiratory samples from 118 patients. The examinations resulted positive in 54 of these (45.8%). *C*_*T*_ values were retrievable for 243 patients irrespectively of patient characteristics, mainly from analysis of BAL-fluid samples (*n* = 192, 79.0%) Table S4 (Additional file 1). Based on the results of the microbiological analyses, 140 patients (47.1%), five of whom were HIV-positive, were diagnosed with PCP (PCP^+^), whereas 116 patients (39.1%) were presumed colonized (PCP^−^) (Figure S1 (Additional file 1)). Epidemiological and clinical characteristics and premorbid iatrogenic exposures of these are summarized in Table S1 and Table S2, respectively (Additional file 1). PCP^+^-patients were generally comparable to the overall population in terms of demographics and predisposition. A tendency of more cardinal symptoms, hypoxia, low lymphocyte counts, elevated lactate dehydrogenase levels, and radiological remarks was noted. The yearly distribution of the three patient categories (PCP^+^, PCP^−^ and “undetermined PCP-status”) is depicted in Fig. 2. There was only one case of PCP in 2006, but the ensuing years saw an increase.

### Complications, management and outcome

From examining the predisposition and clinical characteristics associated with *P. jirovecii-*detection, we went on to investigating the outcome (Table 3). Overall, anti-PCP treatment was instituted to 87.9% of the patients. Almost a third (29.6%) required treatment in an ICU and the same proportion received ventilation support (non-invasive and/or invasive). One hundred twenty-one patients (40.7%) experienced at least one complication, primarily respiratory failure or ARDS. Overall, in-hospital mortality was 21.5%, occurring in 64 patients. Cumulative all-cause 30-, 90- and 180-mortality rates for the study population were 20.2, 33.0 and 39.1% respectively. Accounting for surviving non-participants the rates were lowered resulting in an in-hospital mortality of 17.7% and 30-, 90- and 180-day mortality rates of 16.6, 26.8 and 32.0%, respectively. The clinical course of PCP^+^ patients was broadly similar to the population overall. However, a greater proportion received anti-PCP treatment, intensive care, and ventilation support. Moreover, complication and mortality rates were slightly higher (Table S3, Additional file 1).

### Diagnostic and epidemiological trends

**Fig. 3:**
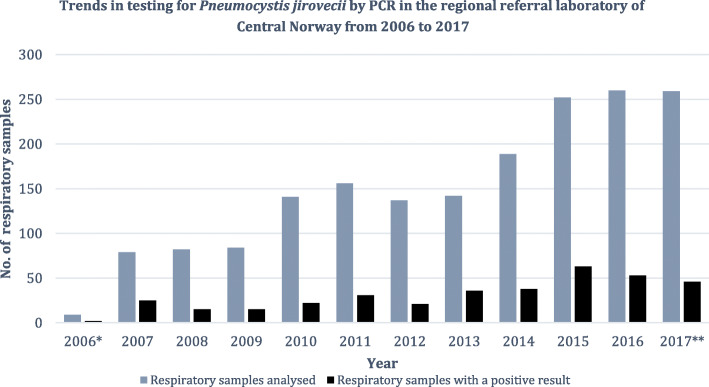
Trends in testing for *Pneumocystis jirovecii* by PCR. Number of respiratory samples referred to the Department of Medical Microbiology of St. Olavs hospital for *P. jirovecii* detection by PCR during the study period (grey columns) and number of respiratory samples resulting positive (black columns). *PCR was introduced in late 2006, and there was a 3.3-fold increase in testing from 2007 to 2017 in our regional referral laboratory. The mean proportion of positive samples (not depicted) was 20.8% (SD 4.7). **In 2017 Molde hospital, a local hospital in the health region, established PCR detection for *P. jirovecii* too. That year an additional 70 respiratory samples were tested at their laboratory, and 20 (28.6%), representing 17 patients, resulted positive (not depicted). PCR, polymerase chain reaction

**Fig. 4:**
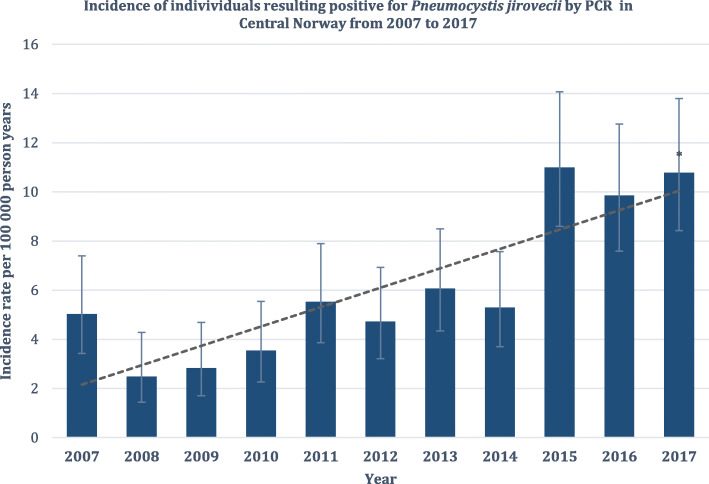
Regional incidence of *Pneumocystis jirovecii* detected by PCR in Central Norway. Estimated incidence rates of individuals resulting positive for *P. jirovecii* by PCR in Central Norway health region (dark columns) with 95% confidence intervals and resulting linear trend (dotted line). PCR was introduced in late 2006 in our regional referral laboratory. Thus, estimates were calculated from 2007. Molde hospital, a local hospital in the health region, established PCR detection for *P. jirovecii* too in 2017. For completeness, individuals resulting positive there were included in the regional incidence estimates for 2017 (*). Regional population counts from Statistic Norway were used to compute the incidence rates. PCR, polymerase chain reaction

Our referral laboratory reported upward trends in molecular testing for *P. jirovecii* during the study period since the introduction of PCR in late 2006. A total of 1790 respiratory samples were referred for PCR analysis; 79 in 2007 compared to 259 in 2017. Accordingly, there was a 3.3-fold increase in analyses from 2007 to 2017. This was accompanied by a 1.8-fold increase in the incidence of samples with a positive PCR result; from 25 in 2007 to 46 in 2017 (Fig. 3). However, the proportion of positive samples remained more or less stable with a mean of 20.8% (SD 4.7) per year. All cases detected within Central Norway gave rise to regional incidence estimates. There were 5.0 cases per 100,000 person years in 2007 compared to 10.8 cases per 100,000 person years in 2017, with an increasing trend during this time interval (Fig. 4).

## Discussion

In the first systematic evaluation of *P. jirovecii* in Norway we observed an apparent increasing incidence of PCP from 2007 to 2017. The vast majority were constituted by patients with other predispositions than HIV-infection, such as haematological and solid cancers, and immunosuppression in the form of corticosteroids in monotherapy or in combination with chemotherapy. Our research confirms the non-specific, thus challenging, clinical presentation of patients with suspected PCP and the association between *P. jirovecii* and high risk of in-hospital mortality.

Several studies have reported upward trends of PCP occurring in non-HIV patients [16–20], including one from Denmark [21]. In contrast, a study from Sweden did not register a rise, in spite of an increasing number of cytotoxic treatments, but that study ended in 2011 [22] which is before we saw a clear increase in our study. The authors proposed a more widespread administration of prophylaxis to patients at risk as the reason for this opposing trend [22]. In our study, only three patients were receiving primary prophylaxis at presentation. However, since only subjects who tested positive for *P. jirovecii* were included in our study population, a selection occurred. As a result, patients receiving prophylaxis without developing PCP, or without undergoing testing during the study period were not included. Nonetheless, our report reveals a gap between patients receiving adequate prophylaxis and those at risk. Concomitantly, a recent Cochrane review showed that trimethoprim/sulfamethoxazole was highly effective in preventing PCP in non-HIV immunocompromised patients with an 85% incidence reduction (95% CI 38 to 96%) and a number needed to treat of 19 patients for PCP prevention [23]. PCP-mortality was also reduced by 83% (95% CI 6 to 97%) without an increase in adverse events [23].

The apparent rise in the PCP-incidence in patients with secondary immunodeficiencies other than AIDS occurs with a concurrent escalation in the administration of immunosuppressants and chemotherapy regimens [1, 24]. Iatrogenic immunosuppression, as well as the impairing effects of the underlying disease itself, are probable explanations for patients developing PCP in this context [25]. Moreover, we report a high prevalence of non-communicable comorbidities which may contribute to the “net state of immunosuppression” [26]. Altogether, this ageing population, cumulating endogenous and exogenous risk factors, may provide favorable conditions for *P. jirovecii* to re-emerge as an important opportunistic pathogen. Nevertheless, since the proportion of positive PCR results remained stable, it is uncertain whether the observations from our region reflects an actual increase in the number of people infected by the fungus, a changed clinical practice, or increased awareness of PCP, namely detection bias.

Reduction of CD4^+^ T cells caused by iatrogenic immunosuppression is the most significant risk factor regarding developing PCP in non-HIV immunocompromised individuals [25]. Systemic corticosteroids are hazardous to lymphocyte proliferation and kinetics, especially in high doses [5]. For the grand majority of our study population, preceding exposure to systemic corticosteroids was a common denominator. We report a wide spectrum of indications, exposure patterns and doses at the time of presentation, as well as a diversity in the co-administered chemotherapy and immunosuppressants. Exposure to systemic corticosteroids preceding development of PCP in heterogeneous non-HIV populations has already been described in several studies [19–21, 27–38]. Even patients receiving systemic corticosteroids in tapering doses are at risk [21]. Moreover, patients with miscellaneous conditions not previously associated to PCP development per se, may develop PCP due to systemic corticosteroids exposure [39]. This was presumably the case for one of our PCP^+^-patients receiving such treatment for statin-induced myositis.

In spite of the widespread use of corticosteroids and their lymphocytotoxic effects, lymphocyte counts were only documented in about half of the patient records (51.2%). Lymphopenia was present in the majority of these (72.1%), and even more prevalent among PCP^+^-patients (93.2%). Neutrophil counts, on the other hand, were present in almost all the records. While neutropenic patients occasionally contract PCP, they do not appear to be unproportionally predisposed to PCP [3], though the risk may depend on the intensity and duration of neutropenic states [40]. Perhaps the missing data in our study, namely the incomplete lymphocyte counts, reflect a certain unawareness and unwariness regarding the impairing effects of immunosuppressants on other cell lines than neutrophils. Raised awareness regarding risk factors would probably lead to more patients receiving primary prophylaxis as well as prompter diagnosis in the case of infection. In fact, early treatment is crucial for the outcome since there appears to be a positive association between treatment delay and mortality [33, 41]. Non-HIV patients seem more susceptible to diagnostic delays in spite of more fulminant onset of symptoms [21, 33, 41].

Regarding outcomes, the in-hospital mortality observed in our study is in the lower range, also among the patients retrospectively classified as PCP^+^. In comparison, it ranges from 15 to 49% for patients without HIV [17, 19, 27, 30, 32, 34–37, 42–45], and increases severely above 50% when ICU admission is required for respiratory failure [29, 46–48]. The differences in mortality may be due to heterogeneity in inclusion criteria in terms of underlying diseases, respiratory samples and diagnostic techniques. A recent meta-analysis reported a pooled overall in-hospital mortality of 30% for patients without HIV [49]. The prognosis of patients with HIV-infection is reportedly better, with mortality ranging from 10 to 20% during the initial infection, but it increases considerably with the need for invasive respiratory support in this population too [3]. In our study, five out of seven patients diagnosed with PCP in the context of AIDS died, resulting in an in-hospital mortality of 71.4%. This sample is too small to draw any conclusions or comparisons but indicates that PCP in HIV-patients is still a serious and potential life-threatening diagnosis, even in an industrialized country like Norway. Indeed, all the HIV-positive individuals fell outside of UNAIDS’ 90–90-90-treatment target for 2020 [50] in spite of high availability of anti-retroviral treatment.

With respect to the distribution of immunocompromising conditions, our cohort is broadly comparable to other reports [27, 30, 32]. In spite of the seemingly increasing incidence, PCP remains a relatively rare disease in non-HIV immunocompromised patients. This is confirmed by our regional incidence estimates for the study period. Importantly, they represent number of people with positive result for *P. jirovecii* by PCR. Hence, the incidence of clinical PCP was likely lower. Fillâtre et al. investigated incidence and risk furtherly; reporting incidence rates of PCP related to non-HIV predisposing conditions over two decades from France [39]. Their results demonstrate an apparent dissimilarity in the risk of contracting PCP within this heterogeneous population, presumably related to the underlying conditions and immunosuppressive treatment [39]. The prevalence of predisposing conditions influences how the risk translates into PCP occurrence. For instance, more patients with rheumatoid arthritis (RA) were assessed for PCP than patients with vasculitidies and connective tissue disorders combined in our cohort. This occurred in spite of RA patients’ inferior risk of contracting PCP compared to the latter group [39]. In developed countries, it is estimated that between 0.5 and 1% of the population suffers from RA [51], whilst vasculitidies and connective tissue diseases are much rarer conditions [52]. This may explain our observations.

To diagnose PCP accurately remains a challenge, even with modern technologies. Herein, positive *P. jirovecii*-PCR was the primary inclusion criterion. To study the epidemiological trends of *P. jirovecii* in Norway, we believe it was important to describe this population as a whole since all the patients were tested on clinical indication and had a high pre-test probability of PCP. In addition, they represented potential candidates for prophylaxis, mostly unidentified at the time, an important aspect to shed light on per se.

DIF microscopy represents an alternative method for case inclusion and is the current gold standard for PCP diagnosis [3]. However, its sensitivity is known to be unacceptably low, especially in populations dominated by HIV-negative individuals [5]. This seemed to be the case in our population as well. Also, false positives may result due to morphologically interchangeable fluorescent material. Lastly, the validity relies on experienced examinators. In light of this, real-time PCR represents a rapid and objective detection tool, though extrapolation of results is confronted by heterogeneity in PCR-target, respiratory samples, quality of DNA-extraction, host-characteristics, quantification methods and so on [53].

Herein, *C*_*T*_ values from semiquantitative real-time PCR analysis and DIF results were collectively used to separate *probable* cases of PCP from those with presumed colonization. Of note, *C*_*T*_ values were not reported in the laboratory information system during the study period. Therefore, *C*_*T*_ values were collected retrospectively from the log of the PCR instruments. Unfortunately, some of the PCR-instruments had been replaced and discarded, and consequently *C*_*T*_ values for samples run on those instruments were lost. Since the retrievability of *C*_*T*_ values depended on which instrument the samples were analyzed, the missing pattern can be considered random and unrelated to patient characteristics. Analysis for beta-D-glucan was not available as a routine assay in our region; thus, such data were unavailable.

Retrospective PCP-classification was a secondary objective to see whether the general trends and characteristics in the overall population were representative. It was performed without considering heterogeneity in respiratory samples, which is a well-known issue [53]. Accordingly, a drawback of this approach is variability in microorganism gradients and volumes across respiratory samples, in addition to intra- and interindividual variability in host-pathogen biology. Collectively, these factors might have resulted in information bias. Yet, regardless of the exact number, the minority of patients with presumed colonization has important implications. Besides the possible role of colonization in chronic diseases, proposed interhuman transmission from individuals harboring *P. jirovecii* organisms is a concern [6].

To our knowledge, this is the largest study undertaken in a Nordic country regarding testing, epidemiology and clinical characteristics of patients assessed for PCP. However, the study design and methodology have several limitations and may provide grounds for biases. Firstly, the study population was sampled from only one region, thus, findings may not be generalizable to other areas. Secondly, the results of this report are based on retrospective case reviews of medical records, a method associated with certain limitations. Foremost, causal claims cannot be made, for instance regarding corticosteroid exposure and risk of contracting PCP. Also, the case review is a qualitative method. Hind-sight bias is likely to affect all retrospective case record reviews in particular [54]. Further, this design does not allow us to examine unavailable patient characteristics, and we rely on the information provided by the health personnel who treated the patients. Thirdly, we were unable to include all alive patients, which might have resulted in selection bias. In spite of the stigma associated with HIV/AIDS, we have little reason to believe that the request for active consent influenced the recruitment of HIV-positive individuals. In fact, the number of HIV-related PCP cases in our cohort were comparable to the estimated incidence in the region according to the national HIV/AIDS surveillance and health reports [7, 55]. Finally, our approach to identify eligible candidates, using positive PCR might have introduced bias as discussed above.

## Conclusions

In conclusion, PCP should always be suspected in susceptible patients manifesting consistent signs and symptoms. Systemic corticosteroid exposure and lymphopenia are dominating risk factors for PCP in non-HIV patients. These appeared to be frequent in our population. Awareness regarding predisposition and the spectrum of onsets, ranging from insidious to fulminant depending on the host’s HIV status, is required to assure a high index of suspicion. Multimodal diagnostics across disciplines are often necessary for precise PCP diagnosing, though biological detection remains fundamental. Here we reveal that PCP is a rare disease in Norway, however the burden of *P. jirovecii* seems to be increasing, especially in non-HIV populations. In light of this, a strategy to increase administration of primary prophylaxis to individuals at risk seems called for.

## Supplementary Information

**Additional file 1.**

## Data Availability

The dataset generated and analyzed during this study are not publicly available due to privacy concerns regarding individual study participants but are available from the corresponding author on reasonable request.

## Abbreviations

AIDS: Acquired immunodeficiency syndrome
AIHA: Autoimmune hemolytic anemia
ARDS: Acute respiratory distress syndrome
CI: Confidence interval
*C*_*T*_: Cycle threshold
CT: Computed tomography
DIF: Direct immunofluorescence microscopy
DMARDs: Disease modifying anti-rheumatic drugs
ECIL: European conference on infections in leukaemia
GVHD: Graft versus host disease
HIV: Human immunodeficiency virus
ICU: Intensive care unit
ITP: Immune thrombocytopenic purpura
PCP: *Pneumocystis* pneumonia
PCR: Polymerase chain reaction
q_n_: n^th^ quartile
RA: Rheumatoid arthritis
REC: Committee for Medical and Health Research Ethics
SD: Standard deviation
