# Supplementary material for: Epidemiological and clinical characteristics of immunocompromised patients infected with Pneumocystis jirovecii in a twelve-year retrospective study from Norway
Source: BMC Infect Dis. 2021 Jul 7;21:659. doi: 10.1186/s12879-021-06144-1 (PMC8262122; doi:10.1186/s12879-021-06144-1)
Supplement: Supplementary file 1 — Additional file 1. [file 12879_2021_6144_MOESM1_ESM.docx]

**Epidemiological and clinical characteristics of immunocompromised patients with positive *Pneumocystis jirovecii*-PCR in a 12-year retrospective study**

Stine Grønseth^1^, Tormod Rogne^2^, Raisa Hannula^3^, Bjørn Olav Åsvold^4,5,6^, Jan Egil Afset^1,7^, Jan Kristian Damås^1,3,8^

1 Department of Clinical and Molecular Medicine, NTNU, Trondheim, Norway

2 Department of Circulation and Medical Imaging, NTNU, Trondheim Norway

3 Department of Infectious Diseases, St. Olavs hospital, Trondheim University Hospital, Norway

4 K.G. Jebsen Center for Genetic Epidemiology, Department of Public Health and Nursing, NTNU, Trondheim, Norway

5 HUNT Research Center, Department of Public Health and Nursing, NTNU, Levanger, Norway

6 Department of Endocrinology, St. Olavs hospital, Trondheim University Hospital, Norway

7 Department of Medical Microbiology, St. Olavs hospital, Trondheim University Hospital, Norway

8 Centre of Molecular Inflammation Research, NTNU, Trondheim, Norway

Keywords:

*Pneumocystis* *jirovecii*, PCP, pneumonia, immunosuppression, immunocompromised

Corresponding author:

Stine Grønseth

Department of Clinical and Molecular Medicine, Faculty of Medicine and Health Sciences

NTNU - Norwegian University of Science and Technology, Trondheim

Postal address: NTNU Department of Clinical and Molecular Medicine, NO-7491 Trondheim, Norway

Phone: +47-93409532; E-mail: stine.gronseth@ntnu.no

| **Table S1 Characterization 140 of patients retrospectively diagnosed with *Pneumocystis* pneumonia (PCP^+^)^a^** | |
| --- | --- |
| **Male sex, n (%)** | 89 (63.6) |
| **Ever smoking, n (%) (n = 136)** | 76 (55.9) |
| **Age (years), median, (q_1_-q_3_)** | 65 (58-73) |
| **Immunosuppressive conditions, n (%)** |  |
| ***Hematological malignancies*** | **48 (34.3)** |
| Non-Hodgkin’s lymphoma | 24 (17.1) |
| Chronic leukemia | 8 (5.7) |
| Acute leukemia | 6 (4.3) |
| Plasma cell disease | 7 (5.0) |
| Hodgkin’s lymphoma | 3 (2.1) |
| ***Solid tumors*** | **41 (29.3)** |
| Lung including pleural membranes | 14 (10.0) |
| Breast | 7 (5.0) |
| Genitourinary tract | 6 (4.3) |
| Other primary tumor^b^ | 7 (5.0) |
| Gastrointestinal tract | 7 (5.0) |
| ***Immunological disorders*** | **17 (12.1)** |
| Connective tissue disorders and vasculitidies | 6 (4.3) |
| Rheumatoid arthritis | 4 (2.9) |
| Miscellaneous^c^ disorders | 7 (5.0) |
| ***Solid organ transplantations*** | **22 (15.7)** |
| Kidney | 18 (12.9) |
| Heart, lung | 4 (2.9) |
| ***Chronic lung diseases*** | **5 (3.6)** |
| Interstitial lung disease or sarcoidosis | 5 (3.6) |
| ***HIV-infection*** | **5 (3.6)** |
| ***Other^d^*** | **2 (1.4)** |
| **Comorbid conditions, n (%)** |  |
| Hypertension | 43 (30.7) |
| Cardiovascular disease | 36 (25.7) |
| Chronic pulmonary disease | 23 (16.4) |
| Diabetes mellitus type 1 or 2 | 20 (14.3) |
| Chronic kidney disease | 19 (13.6) |
| Solid tumor | 14 (10.0) |
| Congestive heart failure | 8 (5.7) |
| Hematological malignancy | 6 (4.3) |
| Rheumatic disease | 4 (2.9) |
| Chronic liver disease | 2 (1.4) |
| **Charlson comorbidity index, n (%)** |  |
| < 4 | 21 (15.0) |
| 4-6 | 59 (42.1) |
| > 6 | 60 (42.9) |

^a^PCP^+^ criteria: i) Positive direct immunofluorescence microscopy and/or ii) Cycle threshold value below 36 on semiquantitative PCR analysis. Patients not fulfilling the criteria were considered colonized (PCP^-^). Patients missing microbiological data were classified with “undetermined PCP-status”.

^b^Other primary tumors include brain tumors (i.e. astroglioma and meningioma), nasopharyngeal carcinoma, adrenal gland tumor, sarcoma, and mesothelioma.

^c^Miscellaneous immunological disorders include hematological disorders (ITP, AIHA), skin disorders, inflammatory diseases of gastrointestinal tract and arthritidies other than rheumatoid arthritis.

^d^Other/miscellaneous immunosuppressive conditions included one patient with no diagnosed condition who had received steroids for suspected autoimmune disorder and one patient with statin-induced myositis treated with corticosteroids.

Abbreviations: AIHA; autoimmune hemolytic anemia, ITP; immune thrombocytopenic purpura, PCR; polymerase chain reaction, PCP; *Pneumocystis* pneumonia

| **Table S2 Premorbid immunosuppression, chemotherapy and corticosteroid exposure among 140 patients retrospectively diagnosed with *Pneumocystis* pneumonia (PCP^+^)^a^** | |
| --- | --- |
| **Immunosuppression/chemotherapy regimens at presentation, n (%)** |  |
| Chemotherapy for hematological malignancy with adjuvant corticosteroids | 31 (22.1) |
| Corticosteroids in monotherapy | 24 (17.1) |
| Graft rejection prophylaxis after solid organ transplantation | 22 (15.7) |
| Chemotherapy for solid malignancy with adjuvant corticosteroids | 16 (11.4) |
| DMARDs with adjuvant corticosteroids | 9 (6.4) |
| Chemotherapy for solid malignancy | 6 (4.3) |
| Chemotherapy for hematological malignancy | 6 (4.3) |
| Corticosteroids and other immunosuppressants^b^ | 4 (2.9) |
| DMARDs in monotherapy | 2 (1.4) |
| Prophylaxis or treatment for GVHD after allogenic stem cell transplantation | 1 (0.7) |
| None | 19 (13.6) |
| **Systemic corticosteroid exposure last 60 days prior to presentation, n (%)** |  |
| Daily | 67 (47.9) |
| Intermittent | 40 (28.6) |
| No exposure to systemic corticosteroids | 31 (22.1) |
| No information | 2 (1.4) |
| **Corticosteroid daily dosage in mg methylprednisolone at presentation, n = 138** |  |
| Median the day of *P. jirovecii* detection (q_1_-q_3_), n = 82 | 10 (6-20) |
| Minimum, maximum | 0,120 |
| **Indications for corticosteroid administration among exposed^c^, n (%)** | |
| Immunosuppression for immunological disorders or graft rejection prophylaxis | 36 (33.6) |
| Chemotherapy | 32 (29.9) |
| Anti-emesis and other oncological indications^d^ | 26 (24.3) |
| Peritumoral oedema in primary and secondary intracranial tumors | 10 (9.3) |
| Hematological and solid malignancies complicated by AIHA or ITP | 5 (4.7) |

^a^PCP^+^ criteria: i) Positive direct immunofluorescence microscopy and/or ii) Cycle threshold value below 36 on semiquantitative PCR analysis. Patients not fulfilling the criteria were considered colonized (PCP^-^). Patients missing microbiological data were classified with “undetermined PCP-status”.

^b^Other immunosuppressants include mycophenolate, azathioprine, cyclophosphamide, calcineurin- and mTOR-inhibitors, cyclosporine and hydroxychloroquine.

^c^107 patients (76.4 %) had known exposure to systemic corticosteroids last 60 days prior to presentation, and proportions are expressed with 107 as denominator. In some cases, corticosteroids were prescribed for more than one indication.

^d^Other oncological indications include peritumoral oedema for patients with extracranial tumors, corticosteroids in combination with radiotherapy, vena cava superior syndrome, medulla compression etc.

Abbreviations: AIHA, autoimmune hemolytic anemia; DMARDs, disease-modifying anti-rheumatic drugs; GVHD, graft-versus-host disease; ITP, immune thrombocytopenic purpura.

| **Table S3 Clinical characteristics, management and outcome among 140 patients retrospectively diagnosed with *Pneumocystis* pneumonia (PCP^+^)^a^** | |
| --- | --- |
| **Symptoms at baseline, n (%)** |  |
| Dyspnea | 104 (74.3) |
| Fever | 107 (76.4) |
| Cough | 85 (60.7) |
| Two symptoms | 106 (75.7) |
| Three symptoms | 52 (37.1) |
| **Objective baseline findings and biochemistry, median (q_1_-q_3_)** |  |
| Oxygen saturation, %, (n = 125)^b^ | 87 (83.4-92.0) |
| Hemoglobin, g/dl, (n = 135) | 10.7 (9.6-11.6) |
| Leukocyte count, x 10^9^/L (n = 137) | 7.6 (4.3-10.0) |
| Neutrophil count, x 10^9^/L (n = 110) | 5.02 (3.0-7.7) |
| Lymphocyte count, x 10^9^/L (n = 73)^c^ | 0.6 (0.3-1.0) |
| Albumin, g/L (n = 106) | 32 (27-36) |
| Lactate dehydrogenase, U/L (n = 86) | 317 (243-439) |
| **Radiological findings, n (%)** |  |
| Remarks on chest X-ray (n = 132) | 116 (87.9) |
| Nodular, linear and/or patchy opacities | 40 (30.3) |
| Focal infiltrates | 12 (9.1) |
| Consolidations | 7 (5.3) |
| Remarks on thoracic CT (n = 118) | 116 (98.3) |
| Ground glass opacities | 97 (82.2) |
| Thickening of interstitial septa | 36 (30.5) |
| Infiltrates | 27 (22.9) |
| Consolidations | 22 (18.6) |
| Lymphadenopathy | 18 (15.3) |
| Bronchiectasis | 8 (6.8) |
| Three-in-bud sign | 6 (5.1) |
| Cysts | 6 (5.1) |
| **Management and complications, n (%)** |  |
| Receiving PCP-directed treatment | 135 (96.4) |
| Antimicrobials for other pathogens^d^ | 92 (65.7) |
| Transferred to an ICU | 50 (35.7) |
| Receiving ventilation support | 46 (33.8) |
| Invasive or invasive and non-invasive | 26 (18.6) |
| Non-invasive only | 20 (14.3) |
| Developing complications | 59 (42.1) |
| Respiratory failure/ARDS | 47 (33.6) |
| Superinfection | 23 (16.4) |
| Hemodynamic failure | 18 (12.9) |
| Renal failure | 16 (11.4) |
| Pneumothorax | 4 (2.9) |
| **Outcome, n (%)** |  |
| In-hospital mortality | 39 (27.9) |
| Cumulative all-cause mortality |  |
| 30-days | 35 (25.0) |
| 90-days | 50 (35.7) |
| 180-days | 58 (41.4) |

^a^PCP^+^ criteria: i) Positive direct immunofluorescence microscopy and/or ii) Cycle threshold value below 36 on semiquantitative PCR analysis. Patients not fulfilling the criteria were considered colonized (PCP^-^). Patients missing microbiological data were classified with “undetermined PCP-status”.

^b^33 patients received supplemental oxygen when oxygen saturation was measured.

^c^Lymphopenia (<1.0 lymphocytes x 10^9^/L) was present among 68 patients (93.2 %) with retrievable lymphocyte counts.

^d^86 patients received antibiotics, 35 patients received antifungals, 16 patients received antivirals other than anti-retrovirals and one patient received anti-tuberculous drugs.

Abbreviations: ARDS, acute respiratory distress syndrome; CT, computed tomography; ICU, intensive care unit; PCP, *Pneumocystis* pneumonia.

| **Table S4 Respiratory samples among study population and microbiological data as basis for *Pneumocystis* pneumonia-status^a^** | | | |
| --- | --- | --- | --- |
| **Respiratory samples** | **Study population overall for reference, n (%)** | **Samples with retrievable results , n (%)^b^** | |
|  |  | **Cycle threshold values semiquantitative PCR analysis** | **Direct immunofluorescence microscopy** |
| Bronchoalveolar lavage fluid | 234 (78.8) | 192 (82.1) | 97 (41.5) |
| Sputum | 44 (14.8) | 37 (84.1) | 13 (29.5) |
| Induced sputum | 9 (3.0) | 7 (77.8) | 4 (44.4) |
| Tracheal aspirate | 5 (1.7) | 4 (80.0) | 3 (60.0) |
| Nasopharyngeal aspirate | 2 (0.7) | 2 (100.0) | 0 (0) |
| Transbronchial biopsy | 1 (0.3) | 1 (100.0) | 0 (0) |
| Biopsy upon autopsy | 2 (0.7) | 0 (0) | 1 (50.0) |
| **Total** | **297 (100)** | **243 (81.8)** | **118 (39.7)** |

^a^ PCP^+^ criteria: i) Positive direct immunofluorescence microscopy and/or ii) Cycle threshold value below 36 on semiquantitative PCR analysis. Patients not fulfilling the criteria were considered colonized (PCP^-^). Patients missing microbiological data were classified as “undetermined PCP-status”.

^b^Cycle threshold values and DIF microscopy results were retrievable for 243 and 118 patients, respectively (%). The total number of the specific respiratory samples (e.g. BAL fluid) is the denominator of the proportions (%) in the two columns to the right.

Abbreviations: BAL, bronchoalveolar lavage; DIF, direct immunofluorescence; PCR, polymerase chain reaction
